# Supplementary figures and images for: Physiological parameters and differential expression analysis of N-phenyl-N′-[6-(2-chlorobenzothiazol)-yl] urea-induced callus of Eucalyptus urophylla × Eucalyptus grandis
Source: PeerJ. 2020 Mar 13;8:e8776. doi: 10.7717/peerj.8776 (PMC7075363; doi:10.7717/peerj.8776)

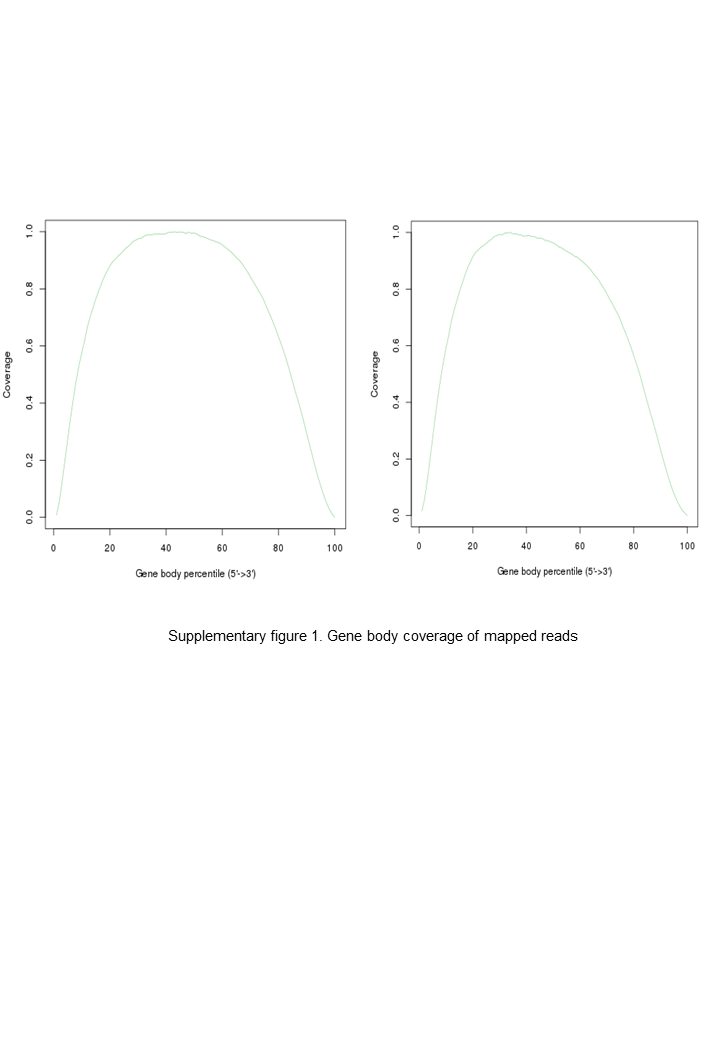

Supplement: Supplemental Information 1 — EC, embryogenic callus; NEC, non-embryogenic callus. [file peerj-08-8776-s001.png]

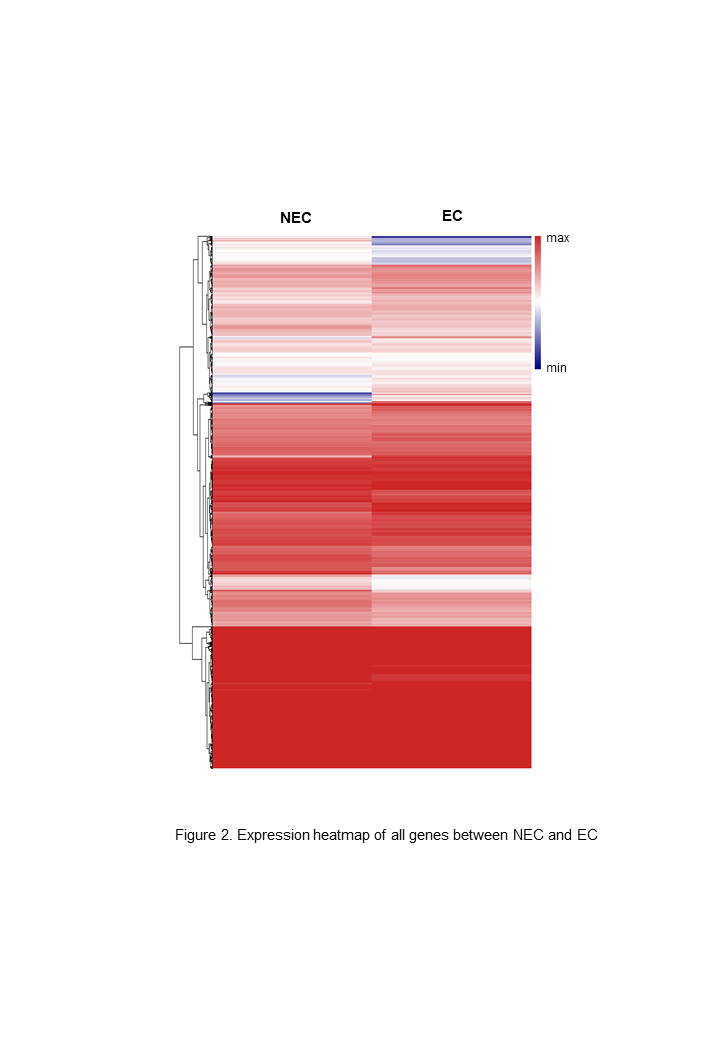

Supplement: Supplemental Information 2 — Red indicates a higher expression level, and blue indicates a lower expression level. Expression calculations are normalized using the CPM value. EC, embryogenic callus; NEC, non-embryogenic callus. [file peerj-08-8776-s002.png]

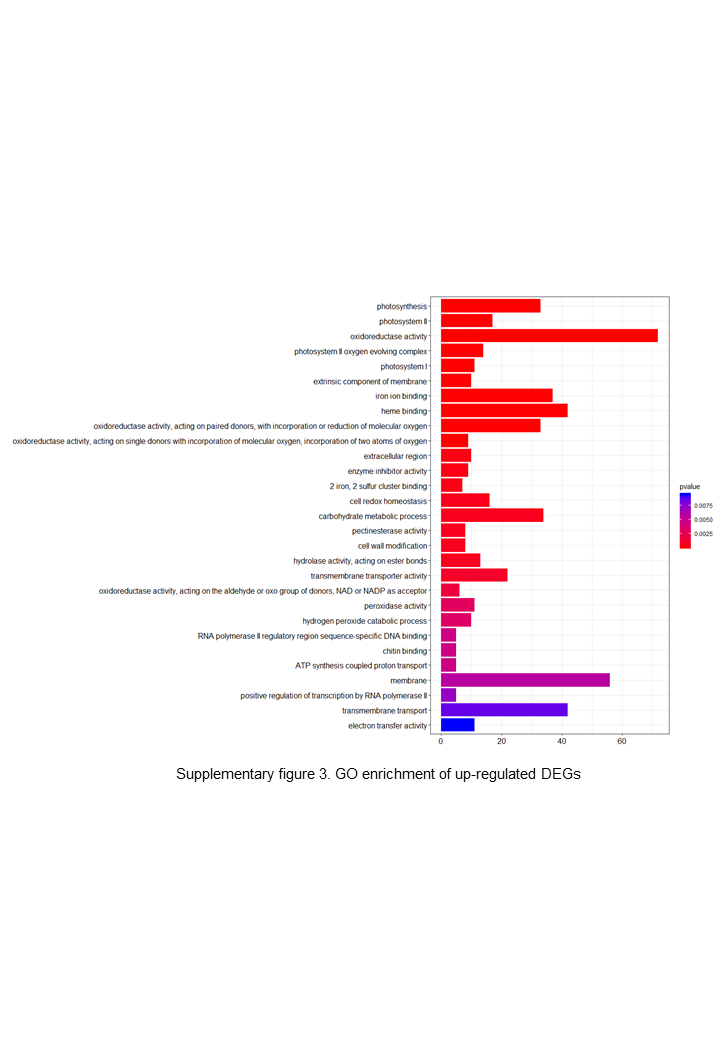

Supplement: Supplemental Information 3 [file peerj-08-8776-s003.png]

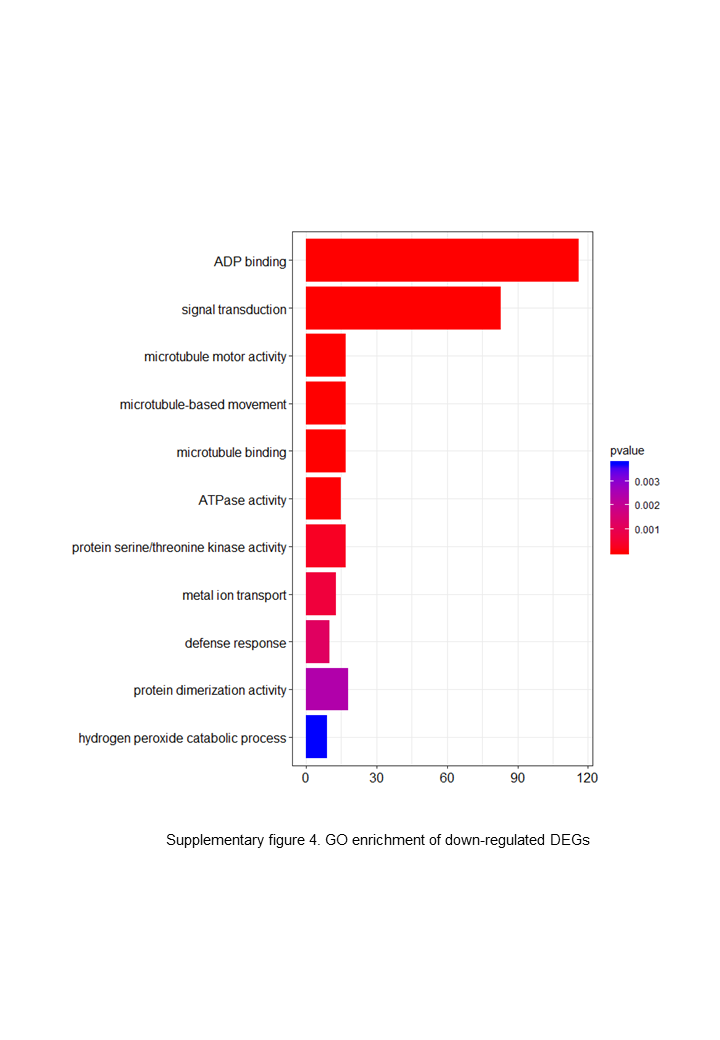

Supplement: Supplemental Information 4 [file peerj-08-8776-s004.png]

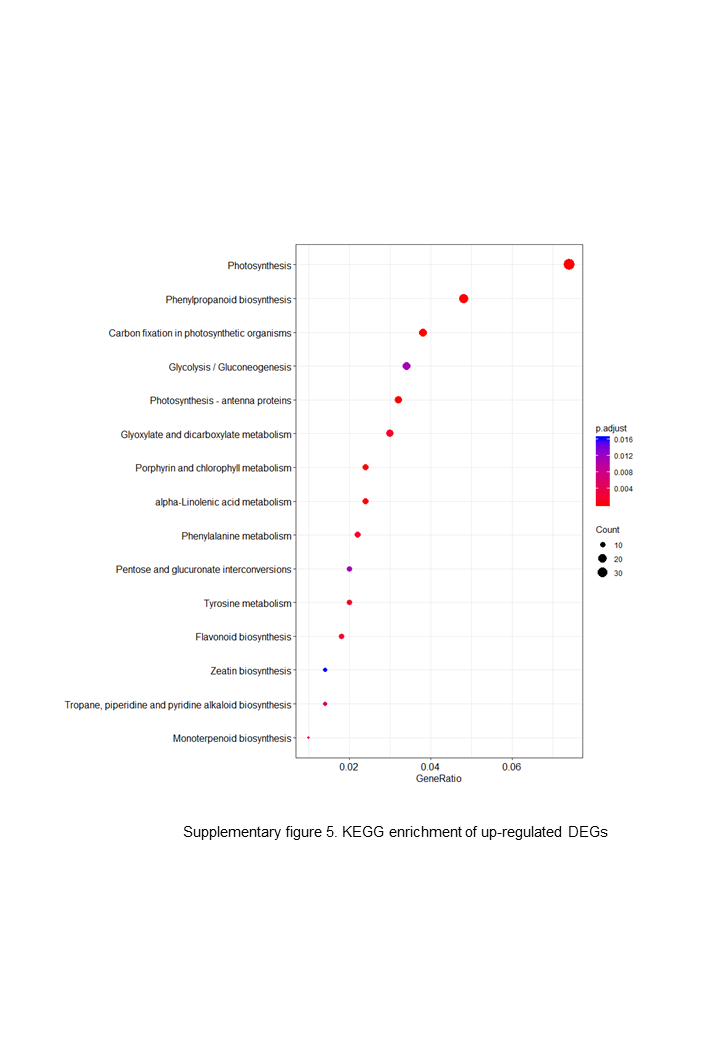

Supplement: Supplemental Information 5 [file peerj-08-8776-s005.png]

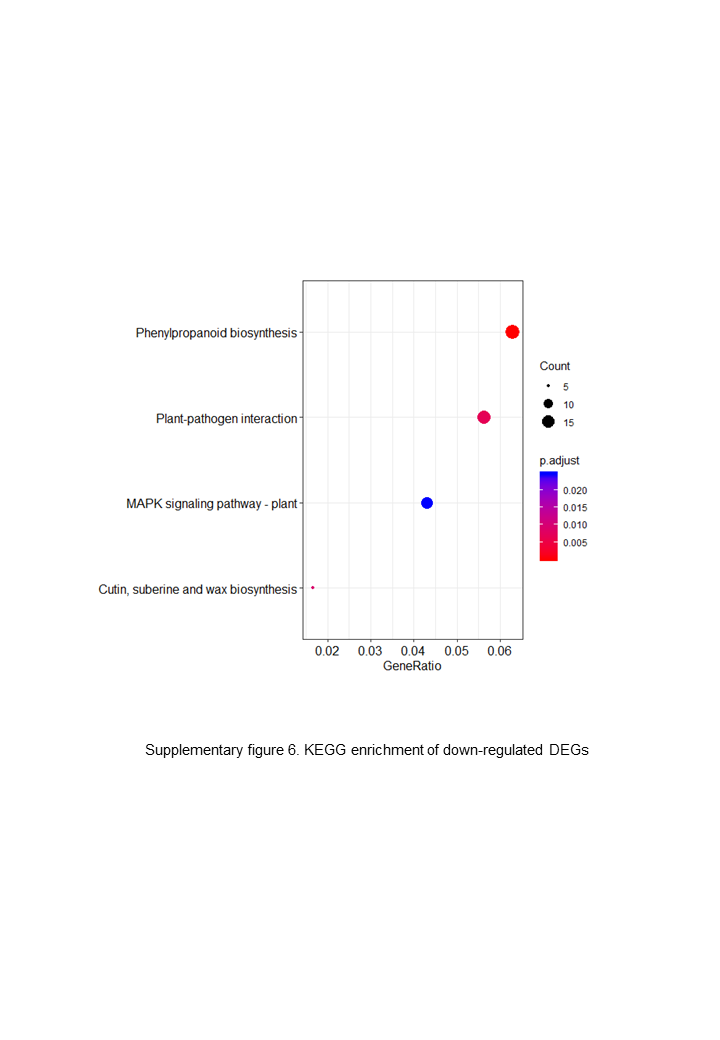

Supplement: Supplemental Information 6 [file peerj-08-8776-s006.png]
