# Supplementary material for: Physiological parameters and differential expression analysis of N-phenyl-N′-[6-(2-chlorobenzothiazol)-yl] urea-induced callus of Eucalyptus urophylla × Eucalyptus grandis
Source: PeerJ. 2020 Mar 13;8:e8776. doi: 10.7717/peerj.8776 (PMC7075363; doi:10.7717/peerj.8776)
Supplement: Supplemental Information 7 [file peerj-08-8776-s007.docx]

Supplementary Table 1. Data summary of transcriptome sequencing.

|  | Non-embryogenic callus | Embryogenic callus |
| --- | --- | --- |
| Total reads | 44,256,994 | 47,888,468 |
| Non primary hits | 4701880 | 11378972 |
| Unmapped reads | 4432947 | 3241173 |
| Mapped reads (ratio) | 35122167 (79.4%) | 33268323 (69.5%) |
| Non-unique map | 2681217 | 5926283 |
| Unique map | 32440950 | 27342040 |
| Non-splice reads | 18771539 | 16018572 |
| Splice reads | 13669411 | 11323468 |
| Reads mapped in proper pairs | 30165864 | 25573714 |
